# Acetylcholine nicotinic receptors play a central role in the modulation of rewarding behaviors by interacting with dopamine transmission: evidence from male rat sexual behavior

**DOI:** 10.1007/s00213-025-06903-x

**Published:** 2025-09-25

**Authors:** Ana Evelia Hernández-Colín, Ana Canseco-Alba, Gabriela Rodríguez-Manzo

**Affiliations:** 1 Departamento de Farmacobiología, Centro de Investigación y de Estudios Avanzados del IPN (Cinvestav-Sede Sur), Calzada de los Tenorios 235, Col. Granjas Coapa, CDMX 14330 Mexico City, Mexico; 2 Dirección de Investigación, Instituto Nacional de Neurología y Neurocirugía “Manuel Velasco Suárez”, Mexico City, Mexico

**Keywords:** Acetylcholine, Nicotinic receptors, Dopamine, Male rat sexual behavior, Sexual satiety, Sexual inhibition, Sexual motivation, Mecamylamine, Natural motivated behaviors

## Abstract

**Rationale:**

Nicotinic acetylcholine receptors (nACh) are involved in the regulation of dopamine (DA) transmission at the mesolimbic (MSL) system, which regulates naturally rewarding behaviors like sexual behavior. Acetylcholine (ACh) and DA maintain a balance in the MSL system, which alteration impacts motivated behaviors. Copulation to satiety produces a sustained activation of the MSL system that triggers the instatement of a long-lasting sexual inhibitory period associated with a decreased sexual motivation.

**Objective:**

To determine if ACh contributes to the establishment and maintenance of the sexual inhibitory period of sexually satiated male rats through the activation of nAChRs.

**Methods:**

Animals copulated to satiety in the presence of different doses of the nAChR antagonist mecamylamine (MEC), systemically administered, to determine nAChR involvement in the establishment of the sexual inhibitory state. The effect of different MEC doses on satiated rats was investigated to determine the role of nAChRs in maintaining the sexual inhibitory state. Combined treatments of MEC with a DA receptor agonist or antagonist were used to determine the possible interaction between DA and ACh.

**Results:**

nAChRs blockade during copulation to satiety interfered with the emergence of the sexual inhibitory state. In sexually satiated rats, nAChR antagonism reversed the sexual inhibition, an effect that was cancelled by a DA receptor antagonist.

**Conclusions:**

ACh is released during copulation to satiety and contributes to the instatement and maintenance of the sexual inhibitory state of sexually satiated rats through the activation of nAChR. DA is involved in the MEC-induced reversal of sexual satiety.

**Supplementary Information:**

The online version contains supplementary material available at 10.1007/s00213-025-06903-x.

## Introduction

Midbrain dopamine (DA) neurons play a key role in reward processing and motivation (Berridge 2012). The DA neurons originating in the ventral tegmental area (VTA) and projecting mainly to the nucleus accumbens (NAcc) and the prefrontal cortex, constitute the mesolimbic (MSL) system, also known as the reward circuit (Ikemoto 2007). Modulation of behaviors that are motivated by natural rewards and their reinforcing properties are mediated by the MSL system (Fortin and Roitman 2018; Kelley 2004; Melis and Argiolas 1995), and the execution of natural rewarding behaviors like feeding (Hernandez and Hoebel 1988), drinking (Young et al. 1992), and copulation (Pfaus et al. 1990) activate the MSL system and induce the release DA in the NAcc, increasing its extracellular concentrations.

Acetylcholine (ACh) is an important component of the MSL system, and it has been proposed that DA and ACh systems operate in a dynamic balance, such that changes in one system modify the activity of the other. It has been found that phasic changes in NAcc cholinergic interneurons (CINs) are time-locked to phasic changes in DA neuron activity (Exley and Cragg 2008). Changes in the MSL DA/ACh balance impact motivated behaviors (Hoebel et al. 2007; Lester et al. 2010; Rice and Craigg 2004). Cholinergic pathways innervate key structures of the MSL system. Thus, the VTA receives cholinergic projections from two mesopontine nuclei, the pedunculopontine tegmental nucleus (PPTg) and the laterodorsal tegmental nucleus (LDTg) (Oakman et al. 1995), both of which modulate DA neuron activity (Picciotto et al. 2012; Yeomans and Baptista 1997), augmenting DA release in the NAcc (Floresco et al. 2003). In turn, in the NAcc an important source of ACh is the small population (≈ 5%) of tonically active CINs that form an extensive network intermingled with dopaminergic varicosities (Kawaguchi et al. 1995). In addition, the NAcc also receives direct cholinergic projections from the LDTg (Dautan et al. 2014).

Both nicotinic and muscarinic cholinergic receptors can affect MSL DA levels; however, nicotinic acetylcholine receptors (nAChR) on DA neuron terminals appear to be particularly relevant to the regulation of motivated behaviors by modulating DA terminal release (Collins et al. 2016; Mark et al. 2011). nAChRs on DA neuron terminals act as a presynaptic filter that modifies NAcc DA release depending on the activity of both the DA neuron itself and the CINs (Exley et al. 2008). DA neuron cell bodies also express nAChRs (Jones et al. 1999), which activation directly excites these neurons and causes DA release in the NAcc (Nisell et al. 1994).

The neuronal nAChRs are pentameric, ligand-gated cation channels. The assembly of different subunits (α2 - α7, α9, α10, and β2 - β10) determines the different receptor subtypes (Taly et al. 2009); however, α4β2* (Klink et al. 2001) and α6β2* heteromeric (Gotti et al. 2010) as well as α7 homomeric nAChRs (Klink et al. 2001) are the main nAChR subtypes involved in the modulation of MSL DA neuron activity in the VTA (Faure et al. 2014). In the NAcc, α6β2* nAChRs are predominant (Exley et al. 2008) and α4β2* nAChRs on DA terminals regulate DA release independently of DA neuron firing (Cachope et al. 2012; Threlfell et al. 2012).

Rewarding behaviors moderately increase ACh extracellular concentrations in the NAcc, while sustained elevated ACh levels in this brain region have been associated with aversive states (Mark et al. 1995; Hoebel et al. 2007) and act as a signal that inhibits appetitive behaviors, as during food satiety (Mark et al. 2011; Hoebel et al. 2007; Rada et al. 2005).

Natural rewarding behaviors like sexual behavior stimulate the MSL system (Kelley and Berridge 2002). In male rats, the mere presence of an inaccessible sexually receptive female as well as copulation both activate the MSL system, increasing NAcc DA release (Damsma et al. 1992). When allowed to freely copulate with a same female, male rats will ejaculate repeatedly -seven successive ejaculations on average- before attaining a state of sexual inactivity known as sexual satiety (Beach and Jordan 1956; Rodríguez-Manzo and Fernández-Guasti 1994). Sustained copulation until satiety triggers the instatement of a reversible, long-lasting sexual inhibitory period (lasting ≈ 72 h) that gradually fades away (Rodríguez-Manzo et al. 2011). Twenty-four hours after copulation to satiety, the sexual inhibition is expressed in two different manners: two-thirds of the sexually satiated animals do not respond to the presence of a receptive female rat, and the other third might copulate until attaining one ejaculation, after which copulation is not resumed (Rodríguez-Manzo and Fernández-Guasti 1994). In vivo microdialysis experiments have shown that the population of non-responsive sexually satiated rats has diminished DA basal levels in the NAcc 24 h after the satiety session and when presented with a receptive female, they do not exhibit the typical elevation in NAcc DA concentration. Interestingly, the systemic administration of the endocannabinoid anandamide, a treatment reversing the sexual inhibition of sexually satiated rats, produces an increase in the blunted NAcc DA basal levels that coincides with the display of sexual behavior (Canseco-Alba et al. 2022). Thus, the sexually satiated male rat appears as a suitable model for the study of the mechanisms involved in the regulation of MSL activity after its intense activation and in the control of NAcc DA concentrations that underlie naturally motivated behaviors.

There is strong evidence supporting the notion that sexual satiety is processed in the MSL system. Thus, direct pharmacological manipulations at the VTA and NAcc reverse the established sexual inhibition of sexually satiated male rats (Canseco-Alba and Rodríguez-Manzo 2016; Garduño-Gutiérrez et al. 2013a; Guadarrama-Bazante and Rodríguez-Manzo 2019; Rodríguez-Manzo and Canseco-Alba 2017). Besides, copulation to satiety triggers the release of endogenous opioids and endocannabinoids at the VTA, which activate mu opioid (Garduño-Gutiérrez et al. 2013b) and CB1 receptors, respectively, and induce glutamatergic plasticity in VTA neurons (Rodríguez-Manzo et al. 2021). Finally, copulation to satiety modifies NAcc DA release (Canseco-Alba et al. 2022). On these bases, we decided to explore the possibility that ACh and DA had a combined role in the modulation of male rat sexual behavior expression in the sexual satiety phenomenon. We hypothesized that copulation to satiety might increase ACh release at the MSL system contributing to the establishment and maintenance of the long-lasting sexual inhibition of sexually satiated rats by modulating NAcc DA levels, through the activation of nAChRs.

In the present study, as a first approach, we pharmacologically explored this notion through the systemic administration of mecamylamine (MEC), a nonspecific, noncompetitive nicotinic receptor antagonist. We tested it in three ways: (1) by blocking nAChR with MEC during copulation to satiety to indirectly determine if ACh is released and plays a role in the instatement of the long-lasting sexual inhibitory state through the activation of nAChRs, (2) by administering MEC to already sexually satiated rats (24 h) to determine if ACh is involved in the maintenance of the sexual inhibitory state through the activation of nAChRs, and (3) by administering combined treatments of MEC with a DA receptor agonist or antagonist to the sexually satiated rats to establish a possible interaction between DA and ACh in the maintenance of the sexual inhibitory state.

## Materials and methods

### Animals

This study used sexually experienced adult male Wistar rats (250–300 g body weight) as experimental subjects and sexually receptive adult female Wistar rats as stimuli for the sexual behavior tests. To render male rats sexually experienced, animals were subjected to five independent sexual behavior tests. The sexually experienced males selected for the study had a stable copulatory pattern, with ejaculation latencies shorter than 15 min and minimal variation among subjects. Sexual receptivity was induced in intact females by the sequential subcutaneous injection of estradiol benzoate (12 µg/rat), followed 19 h later by progesterone (6.0 mg/rat). Females were sexually receptive four hours after the progesterone injection. Animals were housed, eight per cage, under inverted light/dark cycle conditions (12 h/12 h; lights off at 10 AM), at 22 °C, and with free access to food and water. The Local Committee of Ethics on Animal Experimentation (CICUAL) approved all experimental procedures (protocol number 0230 − 16), which followed the regulations established in the Mexican official norm for the use and care of laboratory animals NOM-062-ZOO-1999.

### Sexual behavior recording

Sexual behavior observations were conducted in a room under dim red light during the dark phase of the cycle. Male rats were introduced into polycarbonate cylindrical arenas (50 cm diameter, 60 cm height) and a 5-min adaptation period was allowed before introducing a receptive female. Sexual behavior was recorded, determining the percentage of animals showing the different sexual responses, i.e., mounts (M), intromissions (I), ejaculation (E) and copulation resumption after ejaculation (CR). In those animals achieving ejaculation, the sexual parameters recorded were: intromission latency (IL), time from the introduction of the female into the arena until the appearance of the first intromission; mounts (M), number of mounts preceding ejaculation; intromissions (I), number of intromissions prior to ejaculation; ejaculation latency (EL), time from the first intromission until ejaculation and post-ejaculatory interval (PEI), time elapsing between ejaculation and the appearance of the first intromission of the next copulatory series.

### Sexual satiety paradigm

The sexual satiety paradigm used consisted in allowing the sexually experienced male rats to copulate *ad libitum* with a single sexually receptive female until the accomplishment of the satiety criterion, i.e., permitting a 90-min period to elapse after the last ejaculation without the achievement of another ejaculation. Twenty-four hours later, the same animals were tested for sexual behavior with a new sexually receptive female.

### Locomotor activity test

To rule-out non-specific effects of the drug treatments that could have interfered with sexual behavior execution, the males’ spontaneous locomotor activity was recorded. To this purpose, male rats were placed into an acrylic box (33 × 44 × 20 cm) with the floor divided into 12 squares (12 × 12 cm each), and the number of crossings from one quadrant to another during a 5-min period was registered. Between tests, the cage was cleaned to eliminate trace odors.

### Drugs

All drugs were purchased from Sigma-Aldrich Chem. Co. (St. Louis, Mo, USA). Mecamylamine, a nonselective, non-competitive antagonist of nicotinic acetylcholine receptors was dissolved in distilled water and i.p. injected in a volume of 1 ml/kg, 20 min before the experiments. Haloperidol, a nonselective dopamine receptor antagonist, was dissolved in distilled water adding three drops of ascorbic acid (0.01%), and apomorphine hydrochloride, a nonselective dopamine receptor agonist, was dissolved in saline solution. Both dopaminergic drugs were i.p. injected in a volume of 1 ml/kg, with latencies that are specified in the experimental design. Estradiol benzoate and progesterone were dissolved in sesame oil and subcutaneously administered (1 ml/kg) to the females as described under the animals’ heading.

### Statistical analysis

Comparison of the proportions of sexually satiated rats exhibiting the different sexual behavior responses was conducted with the Fisher *F* test. The specific sexual behavior parameter data did not comply with normal distribution and equal variance and were therefore analyzed with non-parametric statistics. The Kruskal-Wallis ANOVA followed by Dunn’s test was employed. Paired comparisons were conducted with the Mann-Whitney *U* test. Spontaneous ambulation data were normally distributed and were therefore analyzed with a one-way ANOVA followed by the Holm-Sidak test. All statistical analyses were performed with the GraphPad Prism program (version 7).

### Experimental design

Experiment 1. To determine if MEC modified copulatory behavior, six independent groups of sexually experienced male rats (*n* = 8 each) were used to build a dose-response curve; one group was treated with vehicle and the other five received different doses of MEC (0.03–1 mg/kg, − 20 min), and a copulatory series was recorded. Thereafter a locomotor activity test was run.

Experiment 2. To establish the possible participation of the cholinergic system, through the activation of nAChRs, in the development of the sexual inhibitory state that follows copulation to satiety, six independent groups of sexually experienced males (*n* = 8 each) were i.p. injected with vehicle or different MEC doses (0.03–3 mg/kg, − 20 min) and subjected to a copulation to satiety session. Twenty-four hours later the copulatory behavior of the satiated rats was recorded along 90 min.

Experiment 3. To establish the possible participation of the cholinergic system in maintaining the sexual inhibitory state through the activation of nAChRs, five independent groups of sexually satiated rats (*n* = 8 each) were injected during the sexual inhibitory state, i.e., 24 h after copulation to satiety, with vehicle or different MEC doses (1–30 µg/kg, − 20 min) and their sexual activity was recorded during 90 min. After the behavioral observations, the satiated animals were subjected to the locomotor activity test.

Experiments 4 and 5. To establish the possible involvement of the dopaminergic system in the effects of MEC on the copulatory behavior of sexually satiated rats, two additional experiments were run.

In the first one, four independent groups of sexually satiated males (*n* = 8 each) were used. One group received a dose of MEC effective for reversing the sexual inhibitory state (3 µg/kg, − 20 min), a second group was injected with the nonspecific dopamine receptor antagonist haloperidol (HAL), at a dose that lacks effects on copulatory behavior (125 µg/kg, − 30 min) (Rodríguez-Manzo 1999), a third group received the combined treatment of HAL + MEC, and a fourth group the combination of vehicles.

In the second one, another four independent groups of sexually satiated rats (*n* = 8 each) were used. One group received a dose of MEC that was subthreshold for reversing sexual satiety (1 µg/kg, − 30 min), a second group received a dose of the nonspecific dopamine receptor agonist apomorphine (APO), at a dose that is subthreshold for reversing sexual satiety (10 µg/kg, − 15 min) (Canseco-Alba and Rodríguez-Manzo 2019), a third group received the combined treatment of APO + MEC, and the fourth group received the combination of vehicles.

After the sexual behavior recordings, the animals were subjected to the locomotor activity test.

## Results

1. **Effects of MEC on copulation of sexually experienced male rats**
  The different MEC doses tested had little effect on the copulatory behavior of sexually experienced male rats. As shown in Fig. 1, the 0.03 mg/kg MEC dose significantly decreased the intromission number (Kruskal-Wallis ANOVA H_4_ = 12.37, *P* = 0.015; Dunn’s *P* < 0.05), while the 0.1 mg/kg dose produced a statistically significant increase in the duration of the postejaculatory interval (H_4_ = 10.75, *P* = 0.03; Dunn’s *P* < 0.05). The intermediate MEC dose (0.3 mg/kg) decreased the intromission number (Kruskal-Wallis ANOVA H_4_ = 12.37, *P* = 0.015; Dunn’s *P* < 0.01), produced a clear tendency towards a decrease in the already short intromission latency exhibited by the sexually experienced control males and significantly increased the postejaculatory interval (Kruskal-Wallis ANOVA H_4_ = 10.75, *P* = 0.03; Dunn’s *P* < 0.05). By contrast, the highest MEC dose tested (1 mg/kg), significantly increased the intromission latency (Kruskal-Wallis ANOVA H_4_ = 16.36, *P* = 0.003, Dunn’s *P* < 0.01) and produced a clear tendency towards an increase in the rest of the parameters but the intromission number as compared with control sexually experienced males. This MEC dose was also the only one decreasing locomotor activity (data not shown), although without cancelling the display of sexual activity.
  **Fig. 1:**
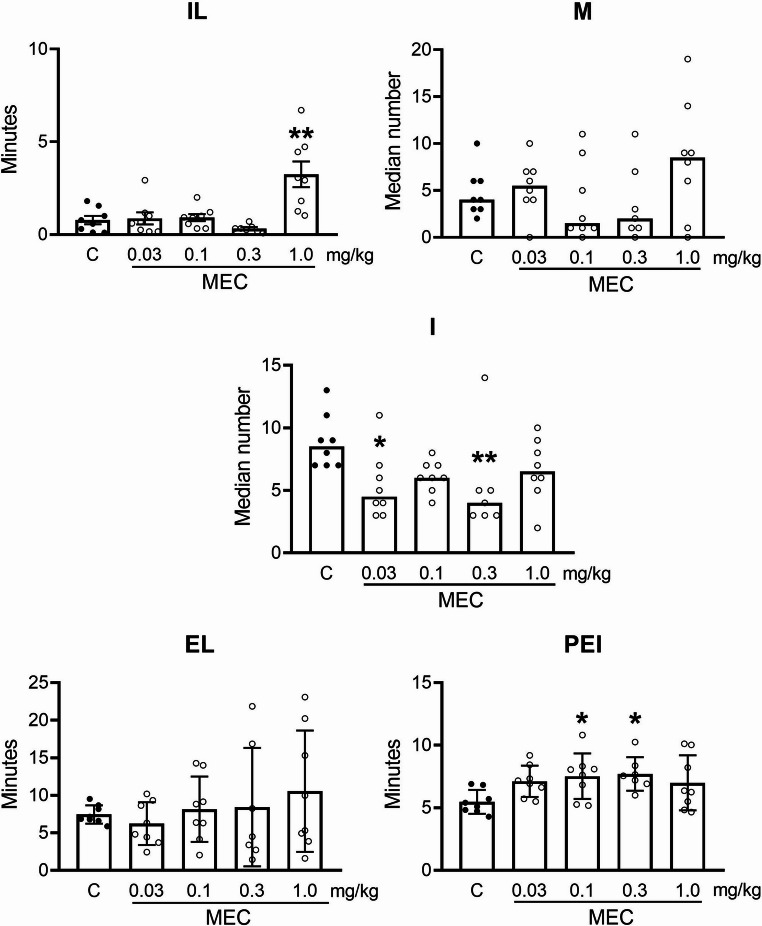
Mecamylamine (MEC) effects on male rat sexual behavior. Specific sexual behavior parameters of sexually experienced male rats treated with different MEC doses (0.03–1.0 mg/kg, i.p.) or its vehicle (C) (*n* = 8 each). Latencies are expressed in minutes, as mean ± SEM, and numbers as medians. Individual data points are included. IL: intromission latency; M: number of mounts; I: number of intromissions; EL: ejaculation latency; PEI: postejaculatory interval. Kruskal-Wallis ANOVA followed by Dunn’s test. *P < 0.05; **P < 0.01 vs. vehicle
2. **Participation of nAChR in the establishment of the sexual inhibition that follows copulation to satiety in male rats**
  Figure 2 shows the percentage of sexually satiated rats that were capable of showing the different sexual behavior responses, 24 h after having copulated to satiety in the presence of different doses of the nAChR antagonist MEC. As can be seen, there was an increase in the proportion of sexually satiated rats, pre-treated with the 0.3 and 1.0 mg/kg MEC doses showing sexual activity during the 24 h test. These increases attained statistical significance for the proportion of animals ejaculating (Fisher *F* test; *P* < 0.01 and *P* < 0.05, respectively) and only the 1.0 mg/kg MEC dose for the proportion of males resuming copulation after ejaculation (Fisher *F* test; *P* < 0.01) as compared to control sexually satiated animals, i.e., this last MEC dose prevented the establishment of the sexual inhibitory state. At the bottom of Fig. 2 the sexual parameters of this last group are shown, which were compared, on the one side, with a historical group of the one third population of sexually satiated males that is capable of ejaculating once 24 h after copulation to satiety and, on the other side, with the parameters of sexually experienced males. Both comparisons showed that the duration of the postejaculatory interval was the only parameter significantly modified by MEC pre-treatment, reducing it when compared with the sexually satiated control animals (Mann-Whitney *U* test, U = 0; *P* < 0.001) and increasing it when compared with sexually experienced rats (Mann-Whitney *U* test, U = 2; *P* < 0.01).
  **Fig. 2:**
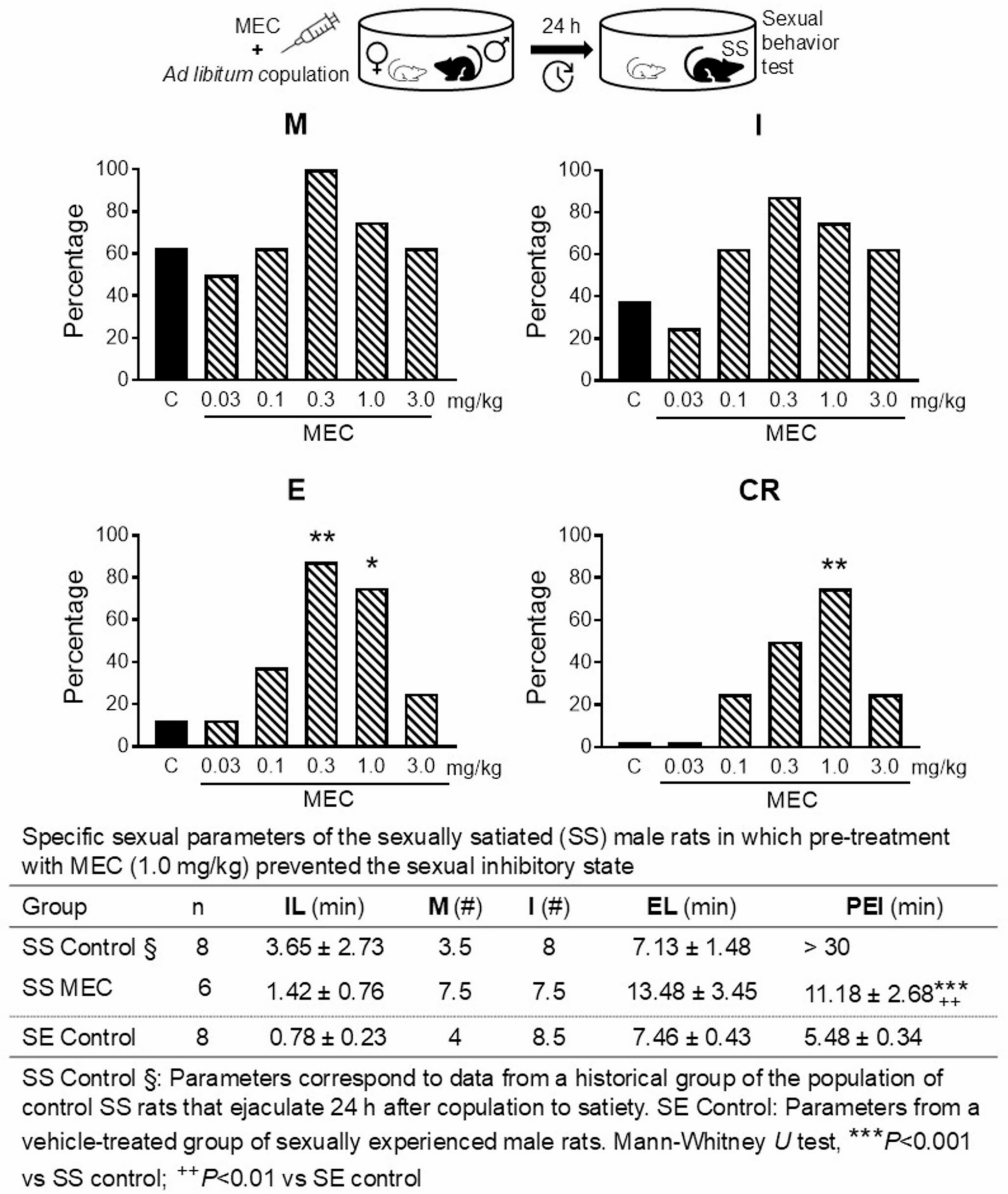
Mecamylamine (**MEC**) actions on the establishment of sexual satiety. The upper part of the figure describes the experimental design used for this experiment. Graphs depict the percentage of male rats that copulated until satiety in the presence of different MEC doses (0.03-3.0 mg/kg, i.p.) or its vehicle (**C**) that showed mounts (**M**), intromissions (**I**), ejaculated (**E**) and resumed copulation after ejaculation (**CR**) 24 h later (*n* = 8 each). Fisher *F* test *P < 0.05; **P < 0.01 vs. vehicle. The specific sexual behavior parameters of the MEC dose preventing the sexual inhibition are presented in the lower part of the figure. Intromission latency (**IL**), number of mounts (**M**), number of intromissions (**I**), ejaculation latency (**EL**), postejaculatory interval (**PEI**)
3. **Participation of nAChR in the maintenance of the sexual inhibitory state of sexually satiated males**
  The proportion of sexually satiated male rats displaying the different sexual behavior elements in response to different doses of MEC administered 24 h after copulation to satiety is shown in Fig. 3. All MEC doses tested increased the proportion of sexually satiated animals showing sexual behavior as compared to the vehicle-treated sexually satiated animals. In fact, with the 3 µg/kg MEC dose all the sexually satiated rats showed mounting behavior, an increase that was statistically significant as compared to the sexually satiated control group (Fisher *F* test, *P* < 0.05). However, only the 3 and 10 µg/kg MEC doses significantly increased the proportion of satiated males that resumed copulation after ejaculation (Fisher *F* test, *P* < 0.05 each), therefore, these two MEC doses reversed sexual satiety. The sexual parameters of these animals appear at the bottom of Fig. 3. Comparison of these parameters with those of the historical group of sexually satiated rats that ejaculates 24 h after satiety shows a significant reduction of the intromission latency only in the group receiving 10 µg/kg MEC (Kruskal-Wallis ANOVA H_2_ = 7.91, *P* = 0.02; Dunn’s *P* < 0.05), as well as decreases in the duration of the postejaculatory interval after both MEC doses (Kruskal-Wallis ANOVA H_2_ = 15.53, *P* ≤ 0.001; Dunn’s *P* < 0.001 for the 10 µg/kg and *P* < 0.05 for the 3 µg/kg MEC doses). When compared with a group of sexually experienced males, a reduction in the intromission number (Kruskal-Wallis ANOVA H_2_ = 8.84, *P* = 0.01; Dunn’s *P* < 0.01) and the ejaculation latency (Kruskal-Wallis ANOVA H_2_ = 6.49, *P* = 0.04; Dunn’s *P* < 0.05) after the 3 µg/kg MEC dose, as well as increases in the duration of the postejaculatory interval after both doses (Kruskal-Wallis ANOVA H_2_ = 14.18, *P* ≤ 0.001; Dunn’s *P* < 0.001 for the 3 µg/kg and *P* < 0.05 for the 10 µg/kg MEC doses) were found.
  **Fig. 3:**
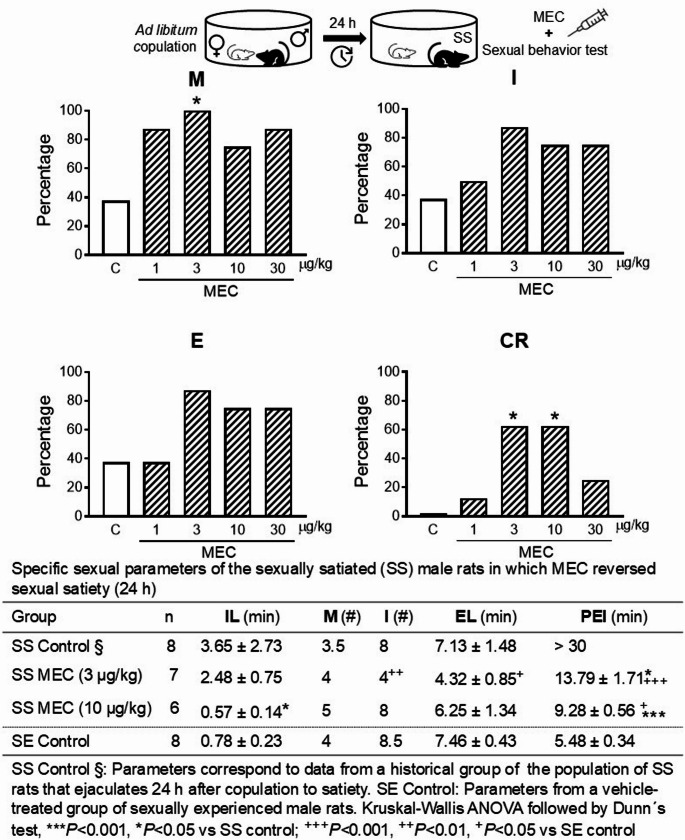
Mecamylamine (**MEC**) actions in sexually satiated rats. The upper part of the figure describes the experimental design used for this experiment. Graphs depict the percentage of sexually satiated male rats showing mounts (**M**), intromissions (**I**), ejaculating (**E**) and resuming copulation after ejaculation (**CR**) in response to the administration of different doses of MEC (1–30 µg/kg, i.p.) or its vehicle (**C**), 24 h after copulation to satiety, (*n* = 8 each). Fisher *F* test *P < 0.05. The lower part of the figure shows the specific sexual behavior parameters of the animals in which MEC reversed sexual satiety. Intromission latency (**IL**), number of mounts (**M**), number of intromissions (**I**), ejaculation latency (**EL**), postejaculatory interval (**PEI**)
4. **Interaction between cholinergic and dopaminergic transmission in the reversal of the sexual inhibitory state of sexually satiated male rats**
  The effects of the combined treatment of MEC with dopaminergic drugs in sexually satiated rats are shown in Fig. 4. Figure 4A depicts the effect of the DA receptor antagonist haloperidol on the MEC-induced reversal of sexual satiety. In these graphs it can be observed that haloperidol (125 µg/kg) per se did not induce sexual behavior expression in the sexually satiated animals, while MEC (3 µg/kg) promoted mounting behavior in 100% of the satiated males, and significantly increased the proportion of these animals showing mounts (M) (Fisher *F* test, *P* < 0.01), intromissions (I), ejaculating (E) and resuming copulation after ejaculation (CR) (Fisher *F* test, *P* < 0.05 each) as compared with sexually satiated animals receiving the combination of vehicles. When combined, haloperidol cancelled the reversal of the sexual inhibition induced by this MEC dose in the sexually satiated rats.
  **Fig. 4:**
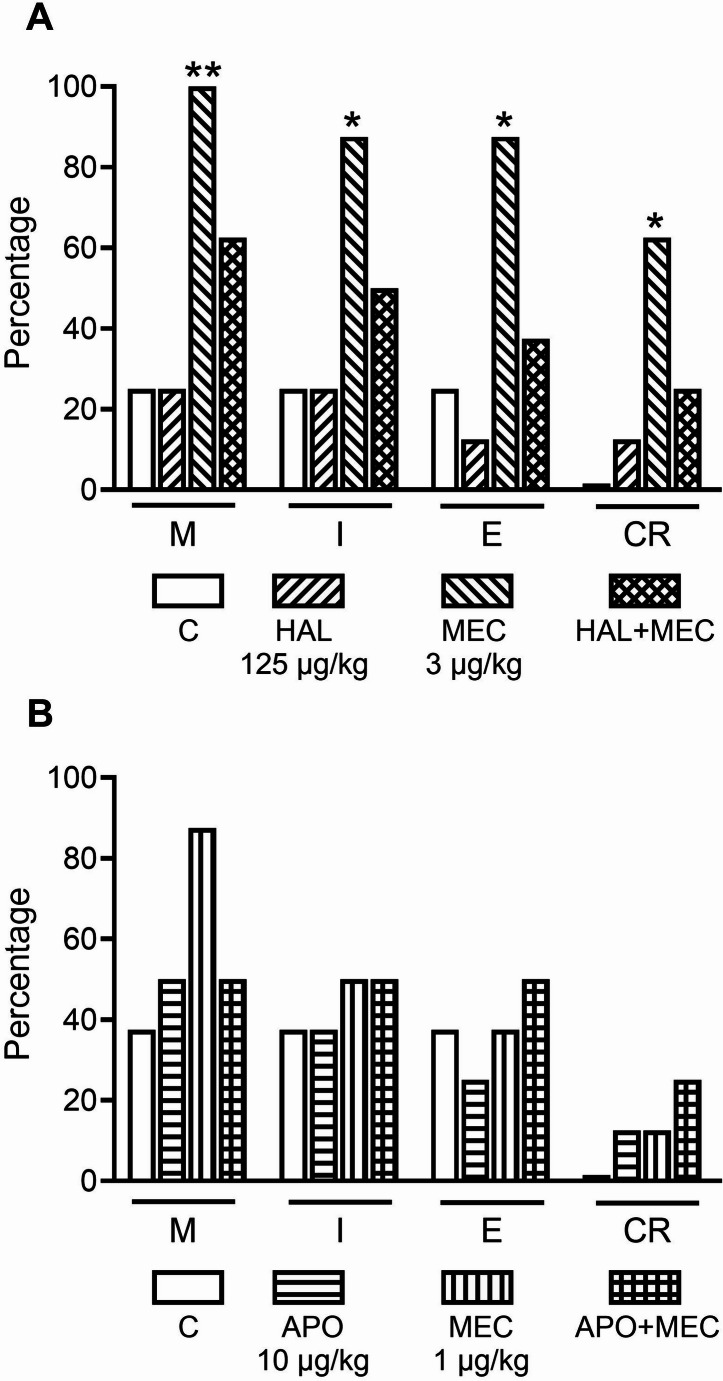
Interaction between mecamylamine (**MEC**) and dopaminergic drugs in the reversal of sexual satiety. (**A**) Percentage of sexually satiated male rats showing mounts (**M**), intromissions (**I**), ejaculation (**E**) and resuming copulation after ejaculation (**CR**) following the i.p. administration of haloperidol (**HAL**) (125 µg/kg), MEC (3.0 µg/kg), HAL + MEC or their vehicles (**C**) (*n* = 8 each). Asterisks over bars indicate comparisons vs. vehicle; Fisher *F* test *P < 0.05, **P < 0.01. (**B**) Percentage of sexually satiated male rats showing the different sexual behavior responses M, I, E and CR after the i.p. administration of apomorphine (**APO**) (10 µg/kg), MEC (1.0 µg/kg), their combination APO + MEC or their vehicles (**C**), (*n* = 8 each). Fisher *F* test, n.s
  Figure 4B shows the effect of the combined injection of sub-effective doses of the DA receptor agonist apomorphine (10 µg/kg) and MEC (1 µg/kg) on the copulatory behavior of sexually satiated male rats. In this case, only MEC augmented the proportion of satiated rats showing mounts but neither drug treatment per se, nor when combined, induced a significant increase in the proportion of sexually satiated animals displaying sexual behavior, evidencing that the combined effect of these drugs was not additive to reverse sexual satiety.
  No differences in spontaneous locomotion of sexually satiated rats (Supplemental tables S1 and S2) were found among groups as compared to control vehicle-treated animals, neither for the sexually satiated animals treated with different MEC doses, nor for those receiving combined treatments with MEC plus the dopaminergic drugs.

## Discussion

The main findings of the present study were as follows: 1) Blockade of nAChRs in sexually experienced male rats moderately affects sexual behavior display in a biphasic manner, showing some facilitatory effects at low antagonist doses; 2) nAChRs are activated during copulation to satiety and contribute to the induction of the long-lasting sexual inhibition that characterizes sexually satiated rats, indirectly evidencing that ACh is released during intense sexual activity; 3) nAChRs are also activated during the sexual inhibitory state, suggesting that ACh contributes to the maintenance of the sexual inhibition of sexually satiated rats; 4) DA is involved in the reversal of sexual satiety produced by nAChR blockade.

The role played by brain nAChRs in male rat sexual behavior has not been studied in depth. Old studies report that systemically administered nicotine (0.1–0.8 mg/kg) decreases the intromission number as the only significant effect (Bignami 1966; Retana-Marquez et al. 1993). There are also studies indirectly assessing the effects of nicotine on human sexuality by analyzing the consequences of tobacco smoking on sexual performance. These studies report erectile dysfunction (Allen and Tostes 2023) and low sex drive (Bhattacharyya et al. 2020) as main effects, together with a negative impact of nicotine on male fertility (Barbagallo et al. 2024). To our knowledge this is the first study analyzing the effect of blocking nAChRs on male rat copulatory behavior. Our results show that in sexually experienced male rats, low doses of the nAChR antagonist MEC tend to facilitate sexual behavior display by reducing several of the already optimal sexual behavior parameters. This is particularly evident for the time it takes for the males to start copulating (intromission latency), which from a mean duration of 47 s in control rats was further reduced to 18 s in response to a specific MEC dose, suggesting a facilitation of sexual motivation. On the other hand, the decrease in the intromission number produced by several MEC doses indicates a reduction in the amount of genital stimulation required to achieve the ejaculatory threshold. In contrast, the highest MEC dose tested produced a clear tendency towards an increase in the values of almost all parameters, evidencing the biphasic profile of MEC acute effects on copulation of sexually experienced rats. This MEC dose reduced the animals’ spontaneous locomotor activity, but this effect did not cancel sexual behavior display.

Endogenous ACh modulates DA terminal release in the NAcc by acting at α4β2*, α6β2* and/or α4α6β2* nAChRs, expressed on DA axon terminals. In the VTA, ACh directly modulates DA neuron activity by acting at α4β2* and α6β2* nAChRs expressed on DA cell bodies (De Kloet et al. 2015). As demonstrated by Rice and Craigg (2004), in the NAcc blockade of β2-containing nAChRs (β2*-nAChR) on DA axons with MEC enhances reward-related DA release. This effect could account for the sexual motivation facilitation observed in response to MEC in the sexually experienced males.

α7-nAChRs indirectly modulate DA release by acting on VTA glutamatergic terminals, where its activation promotes glutamate release, which in turn stimulates neighboring DA neurons (Maex et al. 2014). Thus, blockade of α7-nAChRs with MEC would interfere with this facilitatory mechanism. ACh has a lower affinity for α7-nAChR than for β2*-nAChR. The dose-dependent differential effects of MEC (facilitation or inhibition) on copulation of sexually experienced rats could be related with the nAChR subtype (β2*-nAChR and/or α7-nAChR) that is being blocked with the distinct MEC dose ranges used. Specific experiments should be conducted to explore these possibilities.

In sexually satiated rats, blockade of nAChRs during copulation to satiety promoted a significant increase in the proportion of these animals that were capable of ejaculating 24 h later and, at a specific MEC dose (1 mg/kg), it blocked the instatement of the sexual inhibition that follows copulation to satiety in almost 80% of the animals. In this case, the sexually satiated rats were able to ejaculate and resume copulation after ejaculation 24 h after intense sexual activity, during their expected sexual inhibitory state. These results evidence, on the one side, that ACh is released during copulation to satiety and, on the other, that this neurotransmitter contributes to the establishment of the sexual inhibitory state of sexually satiated males through the activation of nAChRs.

It is well established that the copulation-induced increase in NAcc DA levels persists during repeated ejaculation until satiety (Fiorino et al. 1997; Canseco-Alba et al. 2022), and present data indicate that ACh is released during the same period, although our results do not allow to determine if this takes place in the NAcc. Endogenous ACh release in the NAcc, together with the release of DA has been reported to occur in response to other natural rewarding behaviors like feeding and drinking (Mark et al. 1992). Interestingly, the release of ACh has been associated with the onset of food satiety and considered therefore, to be involved in satiation signaling (Avena et al. 2006; Hoebel et al. 2007; Mark et al. 1992). ACh release might play a similar role in the onset of sexual satiety; however, to confirm this hypothesis it has to be demonstrated that ACh is released in the NAcc of male rats approaching sexual satiety.

Analysis of the specific sexual parameters of the animals in which the instatement of the sexual inhibitory state was prevented, showed that nAChR blockade did not preclude the decay in the sexual performance that characterizes the population of sexually satiated rats that ejaculates during the 24 h test (Rodríguez-Manzo and Fernández-Guasti 1994), except for the fact that MEC pre-treated animals were capable of resuming copulation after ejaculation and therefore exhibited a measurable postejaculatory interval. Together, these data point to the idea that nAChR activation during intense copulation contributes mainly to the decline in sexual motivation that characterizes the sexually satiated rats (Canseco-Alba and Rodríguez-Manzo 2019) but seems not to participate in the deterioration of the sexual performance observed in the sexually satiated animals that are capable of ejaculating 24 h later, as this deterioration was not prevented by MEC pre-treatment.

The contribution of ACh to the establishment of the sexual inhibitory state of sexually satiated rats through the activation of nAChRs is a novel finding. DA neurons, as well as CINs participate in reward-related signaling. DA neurons respond to the presentation of a rewarding stimulus, changing their firing pattern from slow firing rates to burst firing, with a concomitant increase in NAcc DA release (Floresco et al. 2003), while CINs exhibit a brief pause in their tonic activity following reward-related events (Morris et al. 2004). It has been proposed that in the NAcc DA and ACh play opposing roles for approach and avoidance responses, with ACh inhibiting approach and favoring avoidance behaviors through the activation of muscarinic M1 receptors (Hoebel et al. 2007). nAChR activation, by contrast, has been linked to the modulation of DA neuron activity at the VTA (Faure et al. 2014), while in the NAcc the nAChRs on DA terminals act as a synaptic filter to control DA release (Exley and Cragg 2008). It could be thought that in our model MEC pre-treatment modified DA neuron sustained activation during copulation to satiety, by blocking DA cell body β2*-nAChRs in the VTA and increased the neuron activity-dependence of DA release in the NAcc by mimicking terminal β2*-nAChR desensitization. Together, these mechanisms could play a role in interfering with the instatement of the characteristic sexual inhibitory state of rats that copulated to satiety.

The idea of a possible role of nAChR activation in the sexual motivation decline of sexually satiated rats is supported by the finding that MEC was able to reverse sexual satiety in males with an established sexual inhibition (24 h after intense copulation), i.e., it promoted sexual activity until ejaculation and copulation resumption after that ejaculation in a large proportion of the sexually satiated rats. There were two MEC doses effective for reversing the sexual inhibitory state, but only one dose induced an efficient copulatory performance, with sexual parameters that were very similar to those of sexually experienced male rats, except for the duration of the postejaculatory interval. This result suggests that ACh might remain increased during the inhibitory period of sexually satiated males, in contrast with the decreased NAcc DA basal levels characteristic of this period (Canseco-Alba et al. 2022). In line with this possibility, it has been reported in rats that 24 h after bingeing on a sucrose solution (another rewarding stimulus), NAcc DA extracellular levels are reduced, while those of ACh are increased (Avena et al. 2008).

In the NAcc, ACh and DA interact directly at a presynaptic level, with DA being capable of either limiting or promoting ACh release through the activation of D2 and D1 receptors, respectively (DeBoer and Abercrombie 1996). ACh, in turn, can either enhance or inhibit DA release. There is evidence that the nature of this last effect depends on the firing pattern of DA neurons (Zhang and Sulzer 2004). Thus, β2*-nAChR blockade can enhance the reward-related DA release resulting from DA neuron burst firing, but it can also further reduce the decreased DA release that accompanies diminished DA neuron activity (Exley and Cragg 2008). In sexually satiated males, increased ACh levels during the sexual inhibitory state would contribute to maintaining the sexual inhibition.

It has been demonstrated that desensitized β2*-nAChRs on NAcc DA terminals, significantly enhance how DA is released by reward-related burst activity compared to tonic activity (Exley et al. 2008). In the sexually satiated males, blockade of β2*-nAChRs on DA nerve endings could contribute to the reversal of the sexual inhibition of satiated rats by increasing NAcc DA terminal release in the presence of the rewarding stimulus. In support of this possibility is the fact that a small increase in NAcc DA extracellular concentrations coincides with the display of sexual activity in sexually satiated rats after a drug treatment reversing the sexual inhibitory state (Canseco-Alba et al. 2022).

Interestingly, presynaptic nAChRs also control presynaptic activity of CINs (Exley and Cragg 2008). Thus, nAChRs might play a central role in maintaining the ACh/DA dynamic equilibrium at the NAcc for the regulation of the response to rewarding natural stimuli as well as to aversive responses (Hoebel et al. 2007).

In this study, we pharmacologically explored the possible interaction between ACh and DA. Our results showed that the reversal of the sexual inhibition of sexually satiated rats by MEC can be cancelled by pre-treatment with the DA receptor antagonist haloperidol; a result implying that blockade of nAChRs indirectly results in DA receptor activation, probably through an increase in DA release, which might be the final pathway for satiety reversal. Our results also show that co-administration of subeffective doses of the nonselective DA receptor agonist apomorphine and MEC, do not have an additive effect to reverse sexual satiety. This outcome could argue against the hypothesis that the reversal of sexual satiety produced by nAChR blockade is due to an increase in DA release. However, another explanation for this last result relies on the low dose of apomorphine selected, when searching for a dose level that did not reverse satiety per se (Canseco-Alba and Rodríguez-Manzo 2019). The apomorphine dose used for the combination lies within the range reported to activate D2-like presynaptic autoreceptors, both in rodents (Imperato et al. 1988) and humans (De La Fuente-Fernández et al. 2001). Thus, the apomorphine treatment used not only did not activate DA postsynaptic receptors but it might have counteracted the putative MEC-induced DA release by activating terminal D2-like autoreceptors. Specific experiments could clarify this point.

There are several lines of evidence supporting the idea that NAcc DA receptor activation is involved in sexual satiety reversal (Guadarrama-Bazante and Rodríguez-Manzo 2019) and it might represent the final common pathway for diverse drugs, like yohimbine (Rodríguez-Manzo 1999) and the endocannabinoids anandamide and 2-AG (Canseco-Alba and Rodríguez-Manzo 2019). Moreover, it has been demonstrated that the systemic administration of anandamide induces an increase in NAcc DA levels (Solinas et al. 2006), and in sexually satiated rats this increase coincides with the display of sexual behavior (Canseco-Alba et al. 2022). All these data suggest that ACh might produce nAChR-mediated effects on sexual satiety by acting at the MSL system. Experiments determining the effects of local MEC administration in the VTA and NAcc will allow us to confirm or discard this hypothesis.

Sexually satiated rats exhibit an additional feature, simultaneously to the long-lasting sexual inhibition: a generalized hypersensitivity to drug actions (Rodríguez-Manzo et al. 2011). This phenomenon consists in the appearance of drug effects characteristic of high doses at lower dose levels in the sexually satiated males. For instance, the 5-HT1A receptor agonist 8-OH-DPAT induces signs of the serotonergic syndrome at doses lower than those needed to induce them in non-satiated animals. Similarly, the α2-adrenoceptor antagonist yohimbine produces sexual inhibitory effects at lower doses in the satiated males than in non-satiated animals (González-Morales and Rodríguez-Manzo 2020). In the present study, sexually satiated rats were hypersensitive to MEC effects, showing sexual inhibitory responses at doses of MEC that were two orders of magnitude lower than those required to induce them in sexually experienced, i.e. non-satiated, animals. This result encourages the investigation of a possible role of ACh in the induction of the drug hypersensitivity phenomena induced by the intense copulation that triggers sexual satiety.

## Conclusions

The results of this study show that ACh is released during copulation to satiety and plays a role in the instatement and maintenance of the long-lasting sexual behavior inhibition of sexually satiated rats, through the activation of nAChRs. Thus, ACh appears to contribute to the decline in sexual motivation, characteristic of the sexually satiated animals, during the development of the sexual inhibitory state. The participation of nAChRs in the maintenance of the sexual inhibitory state of sexually satiated rats seems to involve dopamine transmission, since its reversal by a nonselective nAChR antagonist includes the activation of DA receptors, suggesting that an increase in DA release might be involved in this last effect. These conclusions are limited by the fact that MEC was systemically administered. Future experiments analyzing the central effects of MEC, specifically at the MSL system, on the sexual satiety phenomenon are granted. This first approximation demonstrates that ACh plays a role in the sexual satiety phenomenon and in the regulation of the sexual motivational tone.

## Supplementary Information

Below is the link to the electronic supplementary material.

Supplementary Material 1

Supplementary Material 2

## Data Availability

The datasets generated during the current study are available from the corresponding author upon reasonable request.
