# Supplementary material for: Acetylcholine nicotinic receptors play a central role in the modulation of rewarding behaviors by interacting with dopamine transmission: evidence from male rat sexual behavior
Source: Psychopharmacology (Berl). 2025 Sep 25;243(6):1499–512. doi: 10.1007/s00213-025-06903-x (PMC13323116; doi:10.1007/s00213-025-06903-x)
Supplement: Supplementary file 1 — Supplementary Material 1 [file 213_2025_6903_MOESM1_ESM.docx]

**Table 1.** Effects of different doses of mecamylamine (MEC) or its vehicle (Veh) on spontaneous locomotor activity of sexually satiated male rats

| **Treatment** | **Dose (μg/kg)** | **Number of counts/5min**  $\bar{\boldsymbol{x}}$**± SEM** |
| --- | --- | --- |
| Veh | 0 | 35.13 ± 1.86 |
| MEC | 1 | 36.75 ± 3.10 |
|  | 3 | 32.63 ± 2.23 |
|  | 10 | 44.75 ± 5.00 |
|  | 30 | 43.25 ± 3.96 |
| One-way ANOVA [F (4,39) = 2.35, non-significant]; n= 8 each | | |
