# Supplementary material for: Acetylcholine nicotinic receptors play a central role in the modulation of rewarding behaviors by interacting with dopamine transmission: evidence from male rat sexual behavior
Source: Psychopharmacology (Berl). 2025 Sep 25;243(6):1499–512. doi: 10.1007/s00213-025-06903-x (PMC13323116; doi:10.1007/s00213-025-06903-x)
Supplement: Supplementary file 2 — Supplementary Material 2 [file 213_2025_6903_MOESM2_ESM.docx]

| **Table 2** Effect of different drug treatments alone or combined on spontaneous locomotor activity of sexually satiated male rats | | |
| --- | --- | --- |
| **Treatment** | **Dose (μg/kg)** | **Number of counts/5min**  $\bar{\boldsymbol{x}}$**± SEM** |
| HAL + MEC Vehicles | 0 | 39.88 ± 2.72 |
| HAL | 125 | 35.63 ± 2.43 |
| MEC | 3 | 32.63 ± 2.23 |
| HAL + MEC | 125 + 3 | 33.13 ± 2.73 |
| APO + MEC Vehicles | 0 | 35.13 ± 1.86 |
| APO | 50 | 38.88 ± 2.07 |
| MEC | 1 | 36.75 ± 3.10 |
| APO + MEC | 50 + 1 | 38.88 ± 1.86 |
| One-way ANOVA for HAL+MEC [F (3,31) = 1.91, non-significant]; one-way ANOVA for APO+MEC [F (3,31) = 0.64, non-significant]; n=8 each  HAL: haloperidol; MEC: mecamylamine; APO: apomorphine | | |
